# Supplementary material for: LOX-1 acts as an N6-methyladenosine-regulated receptor for Helicobacter pylori by binding to the bacterial catalase
Source: Nat Commun. 2024 Jan 22;15:669. doi: 10.1038/s41467-024-44860-9 (PMC10803311; doi:10.1038/s41467-024-44860-9)
Supplement: Supplementary file 1 — Supplementary Information [file 41467_2024_44860_MOESM1_ESM.pdf]

This file contains:

Supplementary Table 1-5, Supplementary Figure 1-12.

Supplementary Tables.

Supplementary Table 1: Demographic data of patients from which *H. pylori*-positive and *H. pylori*-negative gastric samples were harvested.

Patient characteristics	*H. pylori*-positive		*H. pylori*-negative		*p*-values
	n	(%, SD)	n	(%, SD)	
Demographic characteristics					
Female	8	40%	11	55%	0.3422
Male	12	60%	9	45%	
Age (mean, SD)	46.7	16.15	42.6	14.93	0.4097
Severity of gastritis					
Mild	2	10%	3	10.52%	0.54
Moderate	15	75%	16	84.21%	
Severe	3	15%	1	5.26%	

Statistical analysis of patient characteristics was performed using Pearson's chi-squared test ('gender' and 'severity of gastritis') and unpaired two-tailed Student's *t*-test ('Age') with *p*-values less than 0.05 considered statistically significant. Source data are provided as a Source Data file.

Supplementary Table 2: Clinical information of patients from which gastric tissues were harvested before and after antibiotic-based treatment.

Patient ID	Average age	Sex	Pathology Description	Sampling Site	Therapy Type
Patient 1	48.8	M	Gastritis	Antrum	EAC
Patient 2		F	Gastritis	Antrum	Triple therapy
Patient 3		F	Gastritis	Antrum	EAC
Patient 4		M	Gastritis	Antrum	EAC
Patient 5		F	Gastritis	Antrum	EAC
Patient 6		F	Gastritis	Antrum	EAC
Patient 7		M	Gastritis	Antrum	Triple therapy
Patient 8		F	Gastritis	Antrum	NAC
Patient 9		M	Gastritis	Antrum	EAC
Patient 10		F	Gastritis	Antrum	EAC
Patient 11		F	Gastritis	Antrum	EAC
Patient 12		F	Gastritis	Antrum	EAC

M: Male; F: Female; EAC: esomeprazole, amoxicillin and clarithromycin; NAC: N-Acetylcysteine, amoxicillin and clarithromycin; Triple therapy: proton pump inhibitor plus two other types of antibiotics (non-EAC regimen)

Supplementary Table 3: The inclusion and exclusion criteria of patient recruitment.

Inclusion Criteria	Exclusion Criteria
1. Participants aged 18-70 years	1. Individuals with a history of gastric surgery or other major gastrointestinal disorders (e.g., Crohn's disease, ulcerative colitis)
2. Individuals diagnosed with gastritis by endoscopy	2. Pregnant or lactating women
3. Consent obtained from participants in the study	3. Patients with an active bleeding disorder or currently taking anticoagulant medications
	4. Individuals with severe comorbidities (e.g., liver or kidney failure, heart disease) that may affect the study results or patient safety
	5. Participants on antibiotic treatment within the past 2 weeks which may influence *H. pylori* detection
	6. Participants who had received *H. pylori* eradication treatment before

Supplementary Table 4: The primers sequences used in this study.

Primer name	Sequence (5′ to 3′)
H. pylori-F	TTTGTTAGAGAAGATAATGACGGTATCTAAC
H. pylori-R	CATAGGATTTCACACCTGACTGACTATC
LOX-1-F	TTGCCTGGGATTAGTAGTGACC
LOX-1-R	GCTTGCTCTTGTGTTAGGAGGT
LOX-1-MeRIP-F	AGTGCCGACAGCATTGGTG
LOX-1-MeRIP-R	GGAGCAGAGGTATCATAGGAAGC
ACTB-F	CTTCCCAAGAAGGTTCGATTGA
ACTB-R	TCAGACTCTCTTAGGCCAGTTAC
GAPDH-F	ACTTGGCTCCCTTATCTGACC
GAPDH-R	TGTGCAGTGTGAGAAAGGCTT
Il1b-F	GCAACTGTTCCTGAACTCAACT
Il1b-R	ATCTTTTGGGGTCCGTCAACT
Il6-F	TAGTCCTTCCTACCCCAATTTCC
Il6-R	TTGGTCCTTAGCCACTCCTTC
Tnf-F	CCCTCACACTCAGATCATCTTCT
Tnf-R	GCTACGACGTGGGCTACAG
Actb-F	GGCTGTATTCCCCTCCATCG
Actb-R	CCAGTTGGTAACAATGCCATGT
Up_F	ATGGTTAATAAAGATGTGAAACAAAC
Up_R	tagaaaagatcaaaggatcttcCAAAAATCCCATACCATGTCAT
Down_F	atgctcgatgagtttttctaaGAGTAATGTTCCTGAAAGCTTATA
Down_R	CTTGTAACATCCTATCAGGGCTATAG
KanR_F	ATGACATGGTATGGGATTTTTGgaagatcctttgatcttttctac
KanR_R	AAGCTTTCAGGAACATTACTCttagaaaaactcatcgagcatcaaa
katA-BamHI-F	CGGTACCCGGGGATCATGGTTAATAAAGATGTGAAAC
katA-NheI-R	GCTCGCGAAAGCTAGTTACTTTTTCTTTTTTGTGTGG
babA-F	CTTTCCGTGGGGCTTTTTGG

babA-R	GGTGGGGATTTTCACGCCTA
sabA-F	TCAACCGTAAGCAACGCTCT
sabA-R	TGAAACGCCACTGATGACGA
16S rRNA-F	ACGCATAGGTCATGTGCCTC
16S rRNA-R	GTGTCCGTTCACCCTCTCAG
FUT2-F	TCCCCTGGCAGAACTACCA
FUT2-R	GGTGAAGCGGACGTACTCC
FUT3-F	CTGTCCCGCTGTTCAGAGATG
FUT3-R	AGGCGTGACTTAGGGTTGGA

Supplementary Table 5: The siRNA sequences used in this study.

Gene name	Sequence (5′ to 3′)
siMETTL3#1-Sense	CAAGUAUGUUCACUAUGAATT
siMETTL3#1-Antisense	UUCAUAGUGAACAUACUUGTT
siMETTL3#2-Sense	GACUGCUCUUUCCUUAAUATT
siMETTL3#2-Antisense	UAUUAAGGAAAGAGCAGUCTT
siMETTL14#1-Sense	GCAGCACCUCGAUCAUUUATT
siMETTL14#1-Antisense	UAAAUGAUCGAGGUGCUGCTT
siMETTL14#2-Sense	GGUGCCGUGUUAAAUAGCATT
siMETTL14#2-Antisense	UGCUAUUUAACACGGCACCTT
siWTAP#1-Sense	CCCAGCGAUCAACUUGUUUTT
siWTAP#1-Antisense	AAACAAGUUGAUCGCUGGGTC
siWTAP#2-Sense	GGGCAACACAACCGAAGAUTT
siWTAP#2-Antisense	AUCUUCGGUUGUGUUGCCCTT
siLOX-1#1-Sense	CCAUUAUGGUGCUGGGCAUTT
siLOX-1#1-Antisense	AUGCCCAGCACCAUAAUGGTT
siLOX-1#2-Sense	GCAAGACUGGAUCUGGCAUTT
siLOX-1#2-Antisense	AUGCCAGAUCCAGUCUUGCTT
siFUT2#1-Sense	CCATCTACCTGGCCAATTATT
siFUT2#1-Antisense	TAATTGGCCAGGTAGATGGTT
siFUT2#2-Sense	CCACTATATTTCACGTTCATT
siFUT2#2-Antisense	TGAACGTGAAATATAGTGGTT
siFUT3#1-Sense	GACAGATACTTCAATCTCATT
siFUT3#1-Antisense	TGAGATTGAAGTATCTGTCTT
siFUT3#2-Sense	ACTGGGATATCATGTCCAATT
siFUT3#2-Antisense	TTGGACATGATATCCCAGTTT

Supplementary Figures.

Supplementary Figure 1: m^{6}A levels in gastric tissues harvested from patients before and after antibiotic treatment.

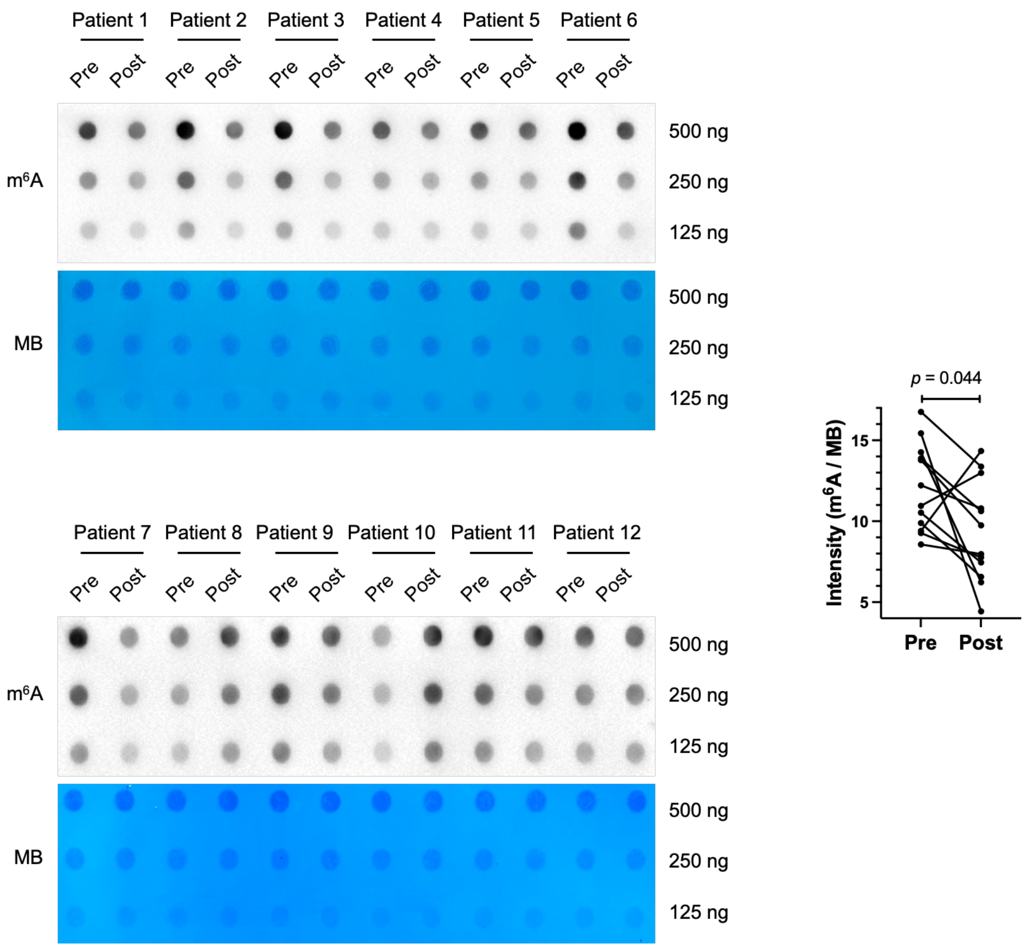

The m^{6}A level of poly(A)$^+$ RNAs in patients' gastric antrum sections (n = 12) before and after treatment with antibiotics was evaluated by m^{6}A dot blot assay. The RNA of each sample was loaded equally with a 2-fold serial dilution. Methylene blue (MB) staining was used as a loading control. The intensity of dots was measured by ImageJ and quantitative analysis was shown as m^{6}A/MB. Quantitative analysis was shown as mean ± SD. Statistical analysis of the data was performed using paired two-tailed Student's *t*-test and the corresponding *p*-values are included in the figure panels. Source data are provided as a Source Data file.

Supplementary Figure 2: m^6A levels in mice with or without *H. pylori* infection.

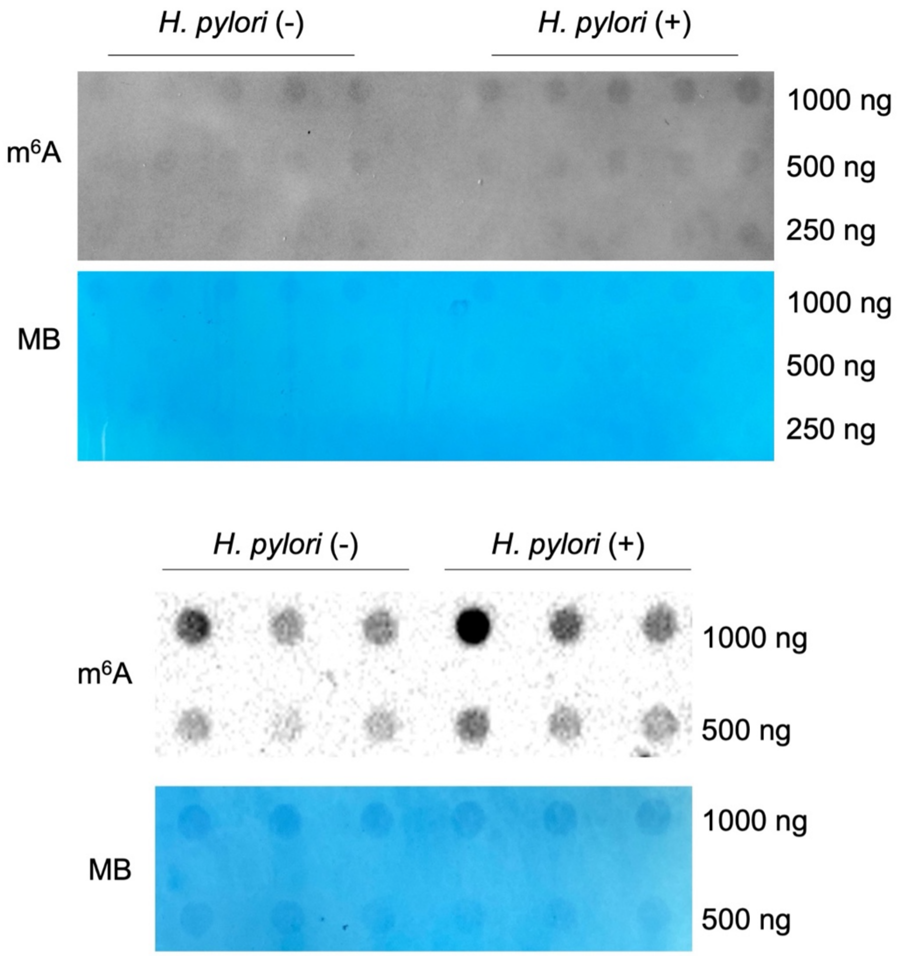

In addition to the 5 samples per group shown in **Fig. 1C**, 8 additional samples per group were shown here. The m^6A level of poly(A)$^+$ RNAs in mouse stomach specimens with (n = 13 in total) or without (n = 13 in total) *H. pylori* colonization was evaluated by m^6A dot blot assay. The RNA of each sample was loaded equally with a 2-fold serial dilution. Methylene blue (MB) staining was used as a loading control. Source data are provided as a Source Data file.

Supplementary Figure 3: Volcano plot of differentially methylated RNA sites (DMRS) of m^{6}A-seq.

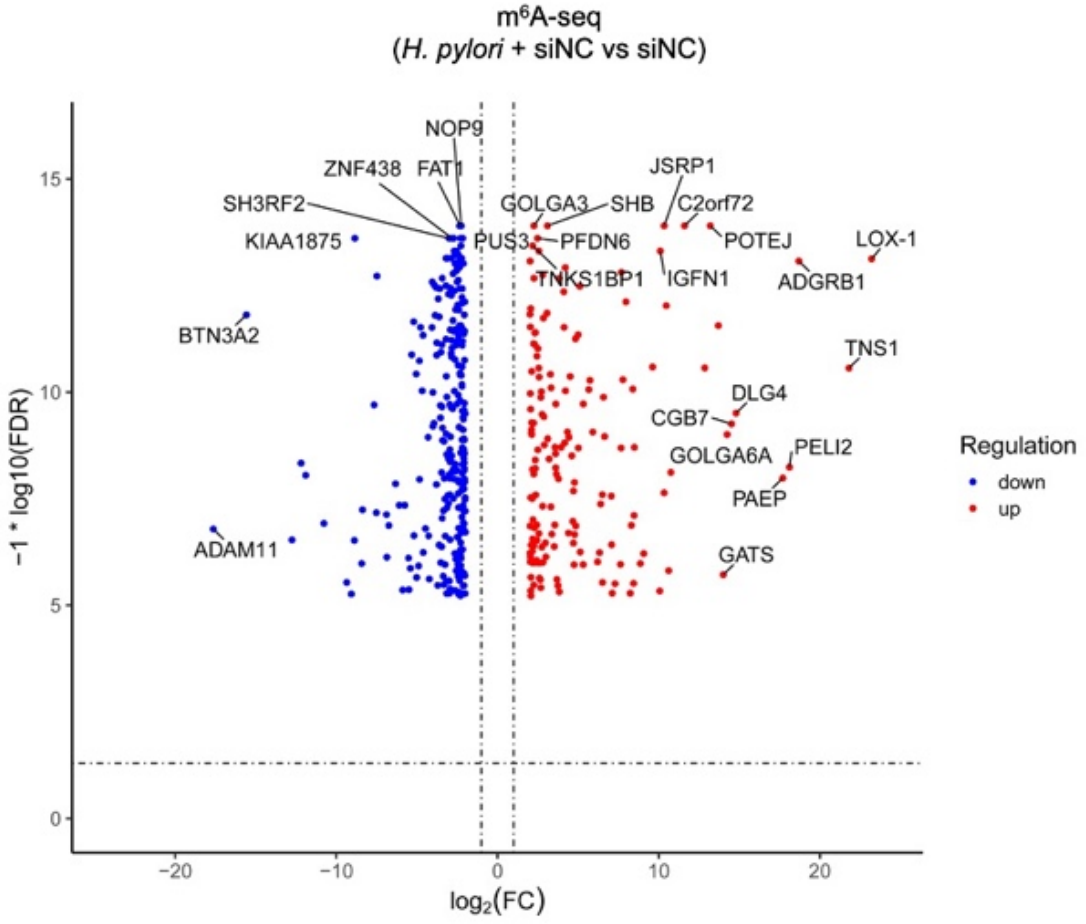

Volcano plot shows the $\log_2$(FC) and -$\log_{10}$(FDR) for all the DMRS (The cut-off of $\log_2$(FC) is larger than 2 and the cut-off of *p*-value is less than 0.0000001). The red dots represent the significantly upregulated genes and the blue dots represent the significantly downregulated genes in the "*H. pylori* + siNC" versus "siNC" comparision. The symbol-marked dots indicate the genes with the most significant FC or FDR. The m^{6}A-seq FC was normalized to the corresponding transcript expression levels in input. FC: fold change; FDR: adjusted *p*-value. Source data are provided as a Source Data file.

Supplementary Figure 4: Volcano plot of differentially expressed genes (DEG) of RNA-seq.

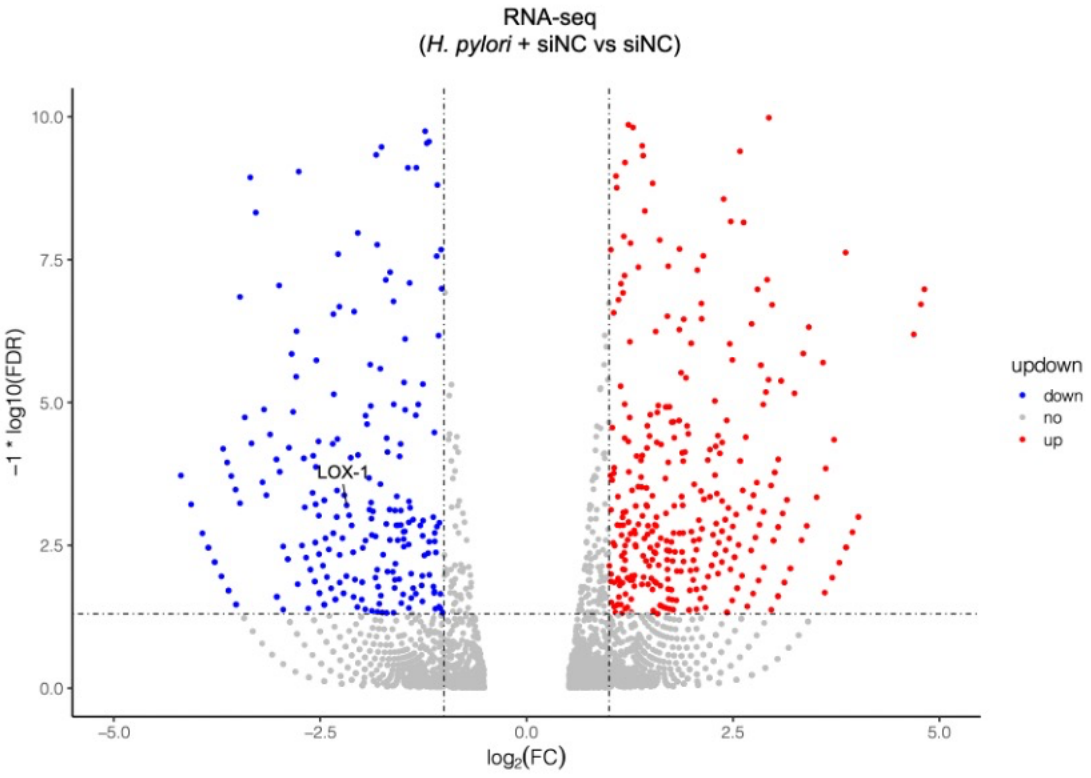

Volcano plot shows the $\log_2$(FC) and $-\log_{10}$(FDR) for all the genes (The cut-off of $\log_2$(FC) is larger than 1 and the cut-off of *p*-value is less than 0.05). The red dots represent the significantly upregulated genes and the blue dots represent the significantly downregulated genes in the "*H. pylori* + siNC" versus "siNC" comparison. FC: fold change; FDR: adjusted *p*-value. Source data are provided as a Source Data file.

Supplementary Figure 5: Histopathological assessment of *Lox-1* WT and *Lox-1*$^{-/-}$ mice gastric sections.

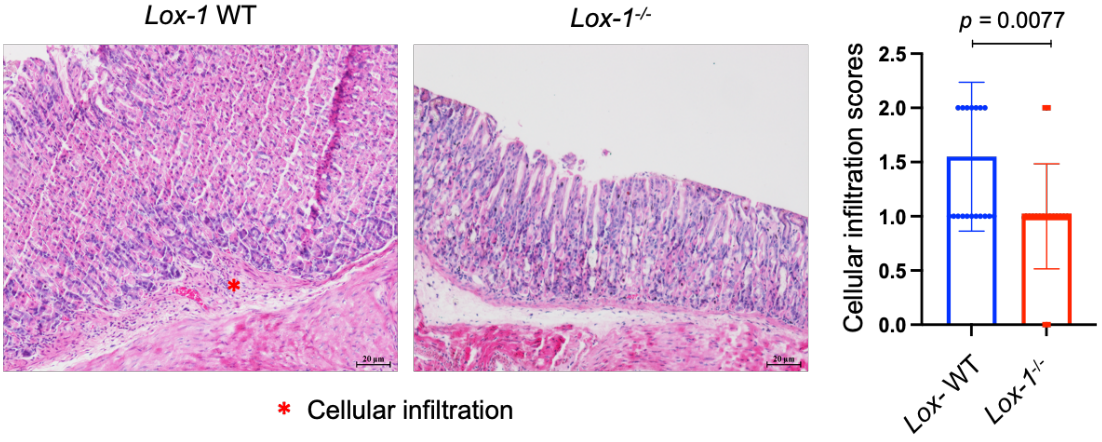

Parafilm-embedded sections of *Lox-1* Wild-type (WT, n = 20 animals) and *Lox-1*$^{-/-}$ mice (n = 18 animals) were stained with hematoxylin and eosin (H&E). Histopathological assessment (cellular infiltration: 0 - 3) was conducted in two separate tissue sections for each animal. Quantitative analysis is shown as mean ± SD. Scale bar, 20 µm. Statistical analysis of the data was performed using unpaired two-tailed Student's *t*-test and the corresponding *p*-values are included in the figure panels. Source data are provided as a Source Data file.

Supplementary Figure 6: The coating of catalase onto fluorescent beads.

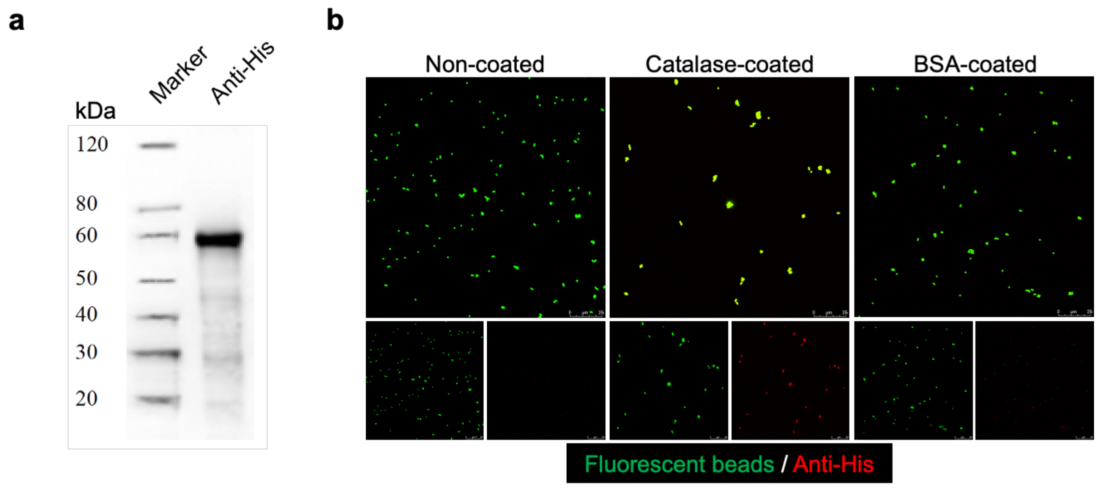

(a) Anti-His antibody was used to examine purified recombinant His-tagged catalase proteins of *H. pylori*; **(b)** The coating of recombinant catalase proteins on fluorescent latex beads was confirmed by immunofluorescence assay with anti-His antibody. Source data are provided as a Source Data file.

Supplementary Figure 7: The construction of isogenic catalase-negative mutant strains.

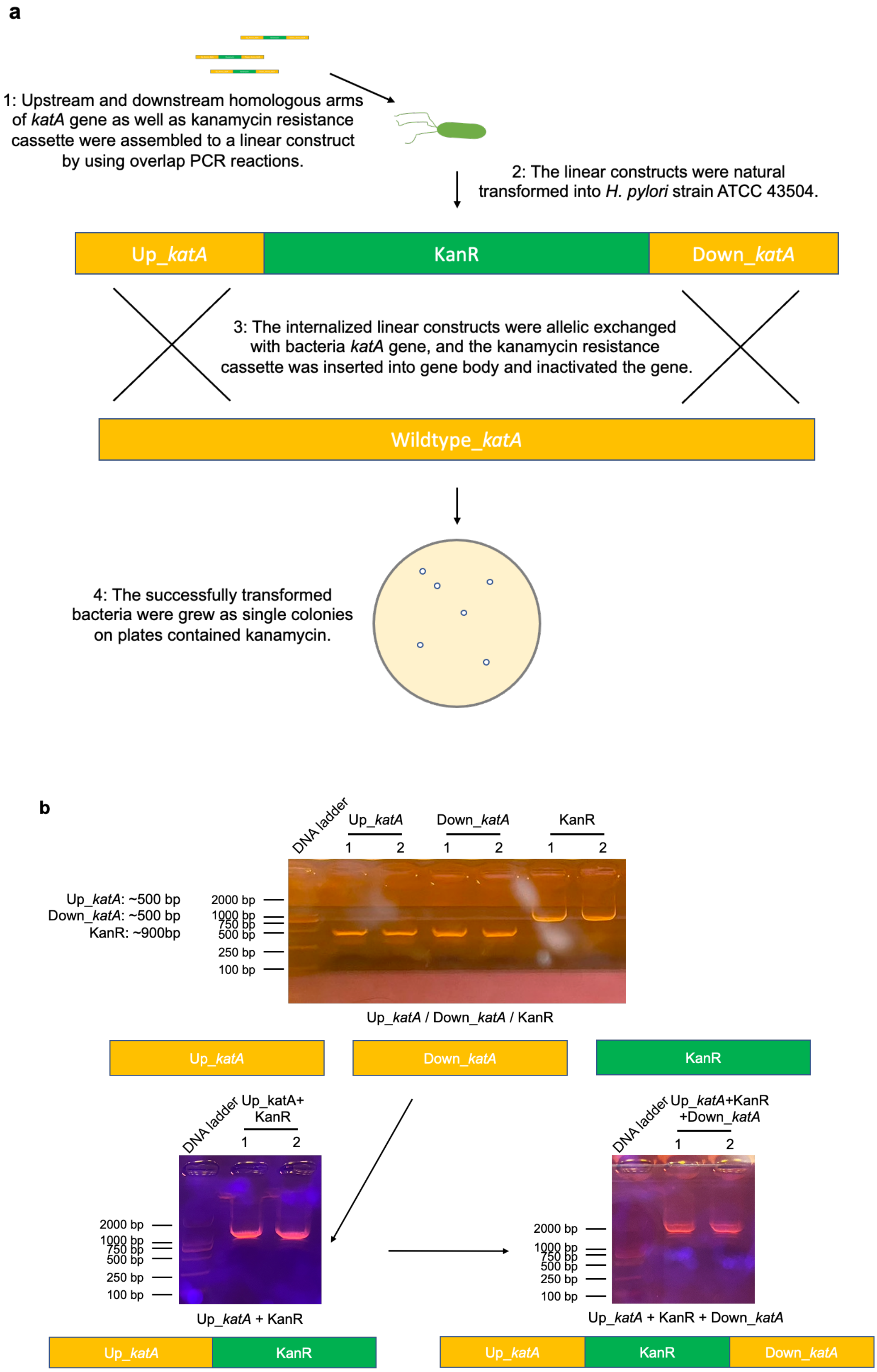

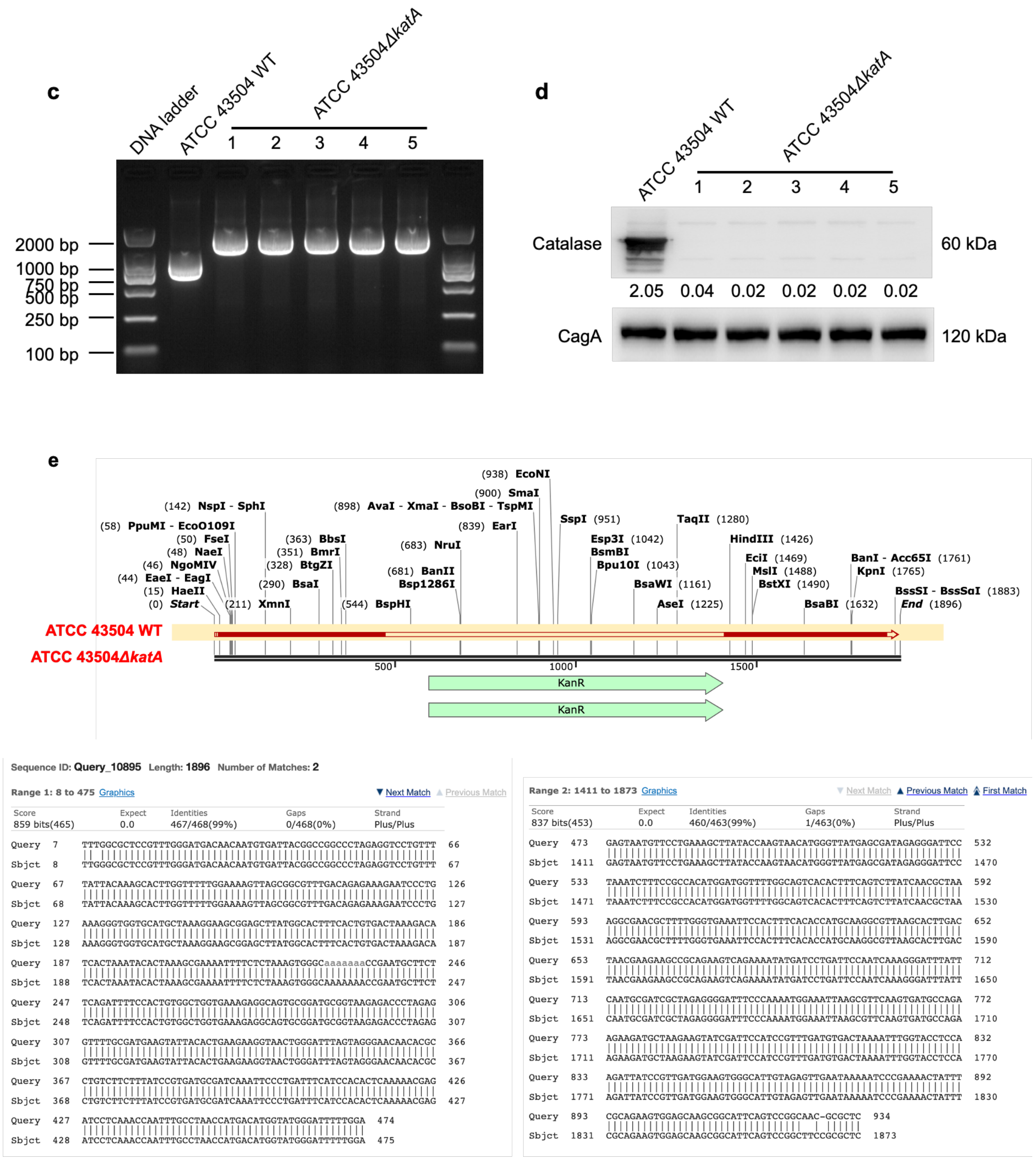

(a) Workflow for the allelic exchange method. **(b)** Three fragments, namely Up_*katA*, Down_*katA* and KanR, were PCR-amplified followed by purififcation from agarose gel. Two sequential overlap PCR reactions were conducted to assemble three PCR products as a linear construct. **(c, d)** Five single colonies on plates containing 50 μg/mL kanamycin were selected to validate the efficiency of allelic exchange. Agarose gel electrophoresis showed that the PCR products of *katA* gene of five colonies were larger than that of the wild type, indicating that *katA* gene was successfully replaced by the linear construct. The results of Western blots showed that no signal of catalase

could be detected in the five colonies, confirming that the selected colonies were catalase-negative mutants. *H. pylori* CagA was used as an internal control. **(e)** The aligment of sequencing data confirmed that *katA* gene was allelic-exchanged by the linear construct containing the kanamycin resistant cassette (KanR) in the selected colonies. Source data are provided as a Source Data file.

Supplementary Figure 8: The genetic complementation of *katA*.

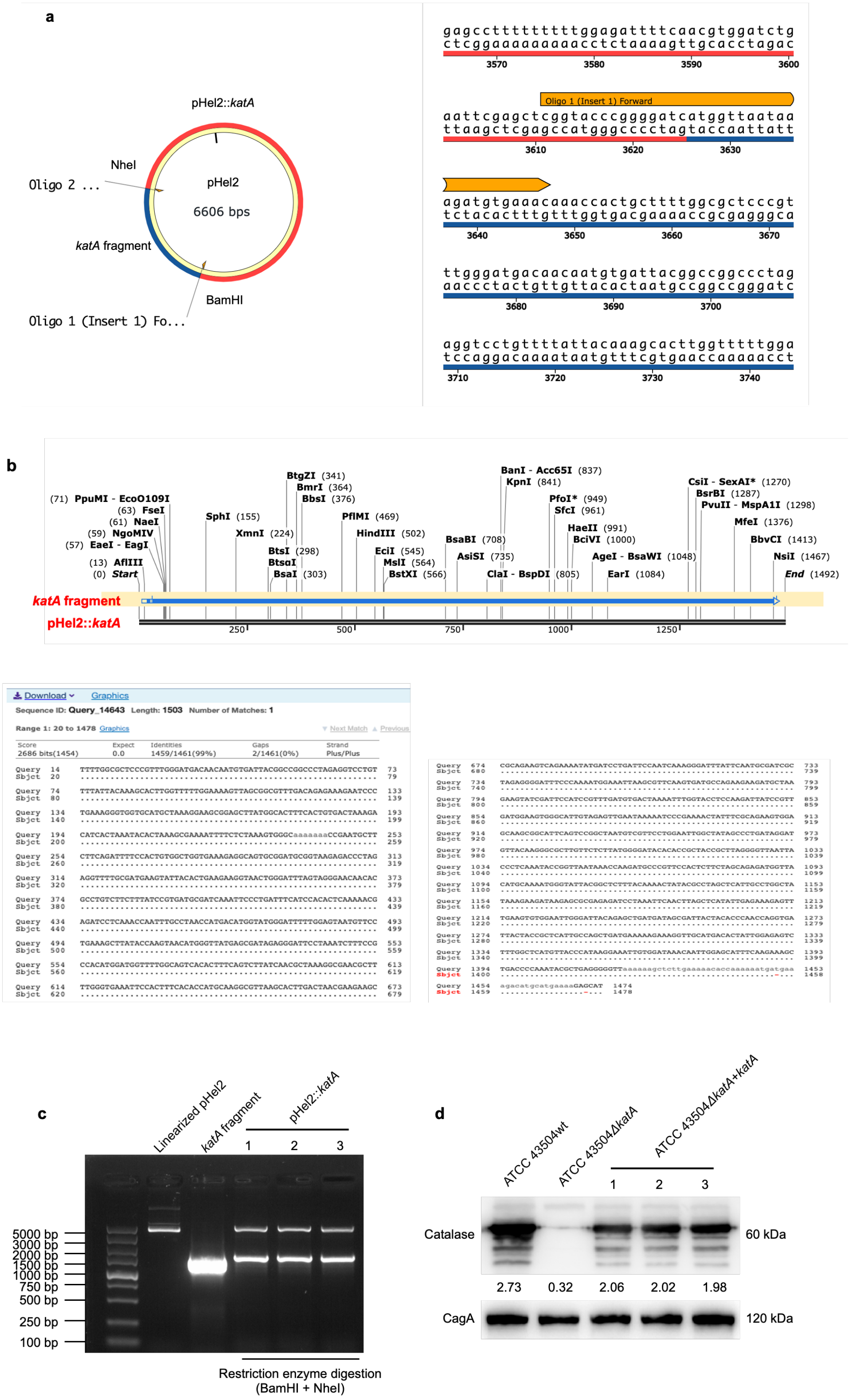

(a) The design for construction of pHel2::*katA* using Takara In-Fusion primer design tool (https://takara.teselagen.com/#/DesignPage). **(b)** The sequencing data of

pHel2::*katA* was aligned to the query sequence of *katA* fragment. **(c)** The recombinant vector pHel2::*katA* was digested with two restriction enzymes, BamHI and NheI, followed by agarose gel electrophoresis to confirm the insertion of *katA* fragment. Linearized pHel2 and purified *katA* fragment were used as control. **(d)** The recombinant vector pHel2::*katA* was electroporated into *ΔkatA* mutant. Western blots for detecting catalase was performed to validate the efficiency of genetic complementation of *katA* in mutant strain. *H. pylori* CagA was used as an internal control. Source data are provided as a Source Data file.

Supplementary Figure 9: Knockout of catalase only affected catalase but not other outer membrane proteins of *H. pylori*.

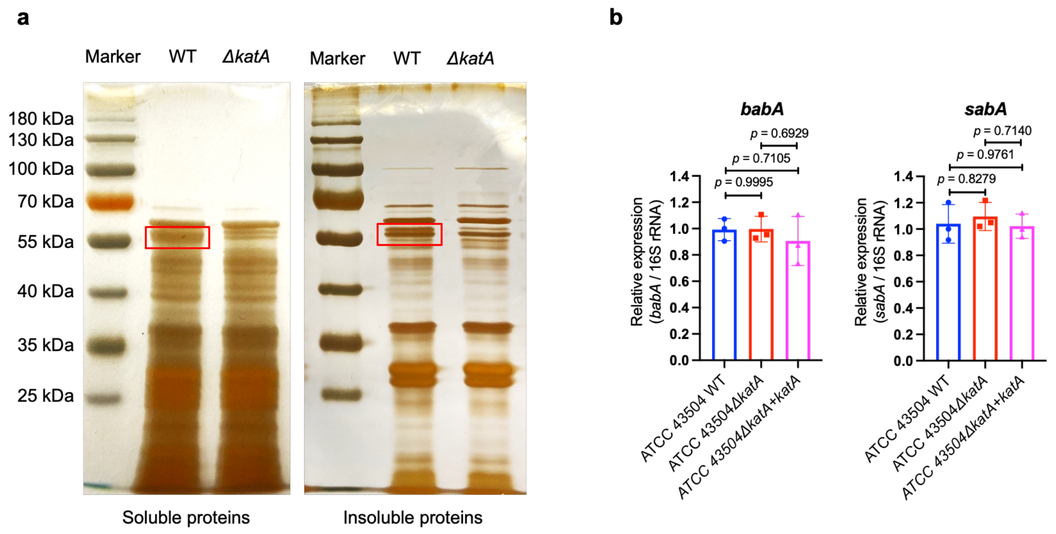

(a) Overall soluble and insoluble bacterial proteins were extracted from ATCC 43504 wild-type and *ΔkatA* strains, and analyzed with silver staining. Molecelar size of catalase is 58 kDa. **(b)** qRT-PCR was performed to detect the expression of *babA* and *sabA* in ATCC 43504 wild-type, *ΔkatA* and *ΔkatA*+*katA* strains. *H. pylori* 16S rRNA was used as an internal control (n = 3 clones for each group). Quantitative data are shown as means ± SD. Statistical analysis of the data was performed using one-way ANOVA followed by Tukey’s multiple comparison tests with adjustments and the corresponding *p*-values are included in the figure panels. The statistical significance of the data was calculated from one of three independent experiments with similar results. Source data are provided as a Source Data file.

Supplementary Figure 10: *H. pylori* strains were labelled with FITC.

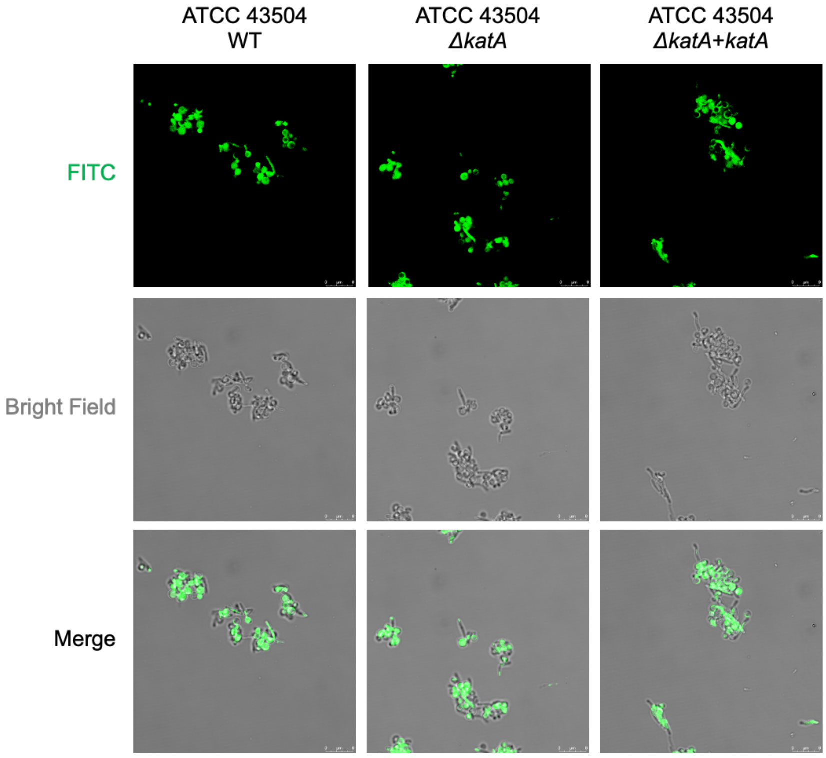

ATCC 43504 wild type, *ΔkatA* and *ΔkatA+katA* strains were labelled by FITC. No obvious difference in morphology or labelling efficiency was observed among the three strains.

Supplementary Figure 11: Pre-heating human normal stomach tissue array at 60°C did not change the trend of adhesion level of ATCC 43504 wild-type, *ΔkatA* and *ΔkatA+katA* strains.

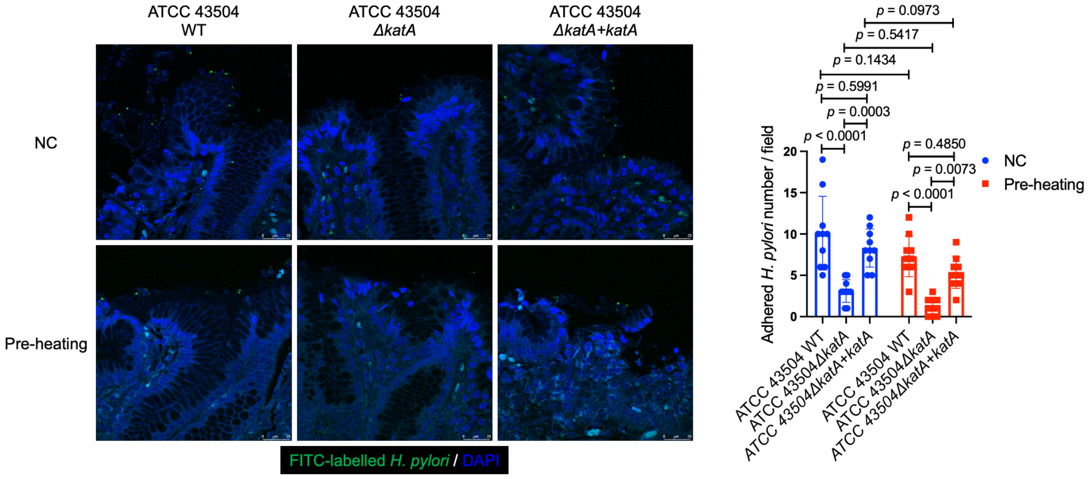

FITC-labelled *H. pylori* strains (wild-type, *ΔkatA* and *ΔkatA+katA* strains) were respectively incubated with human gastric sections with or without pre-heating at 60°C. Ten visual fields of each group were randomly selected to count the number of adhered *H. pylori*. Scale bar, 25 μm. Quantitative data are shown as means ± SD. Statistical analysis of the data was performed using one-way ANOVA followed by Tukey's multiple comparison tests with adjustments and the corresponding *p*-values are included in the figure panels. Source data are provided as a Source Data file.

Supplementary Figure 12: Detection of *babA* expression in *H. pylori* strains and Lewis^b expression in HFE145 cells.

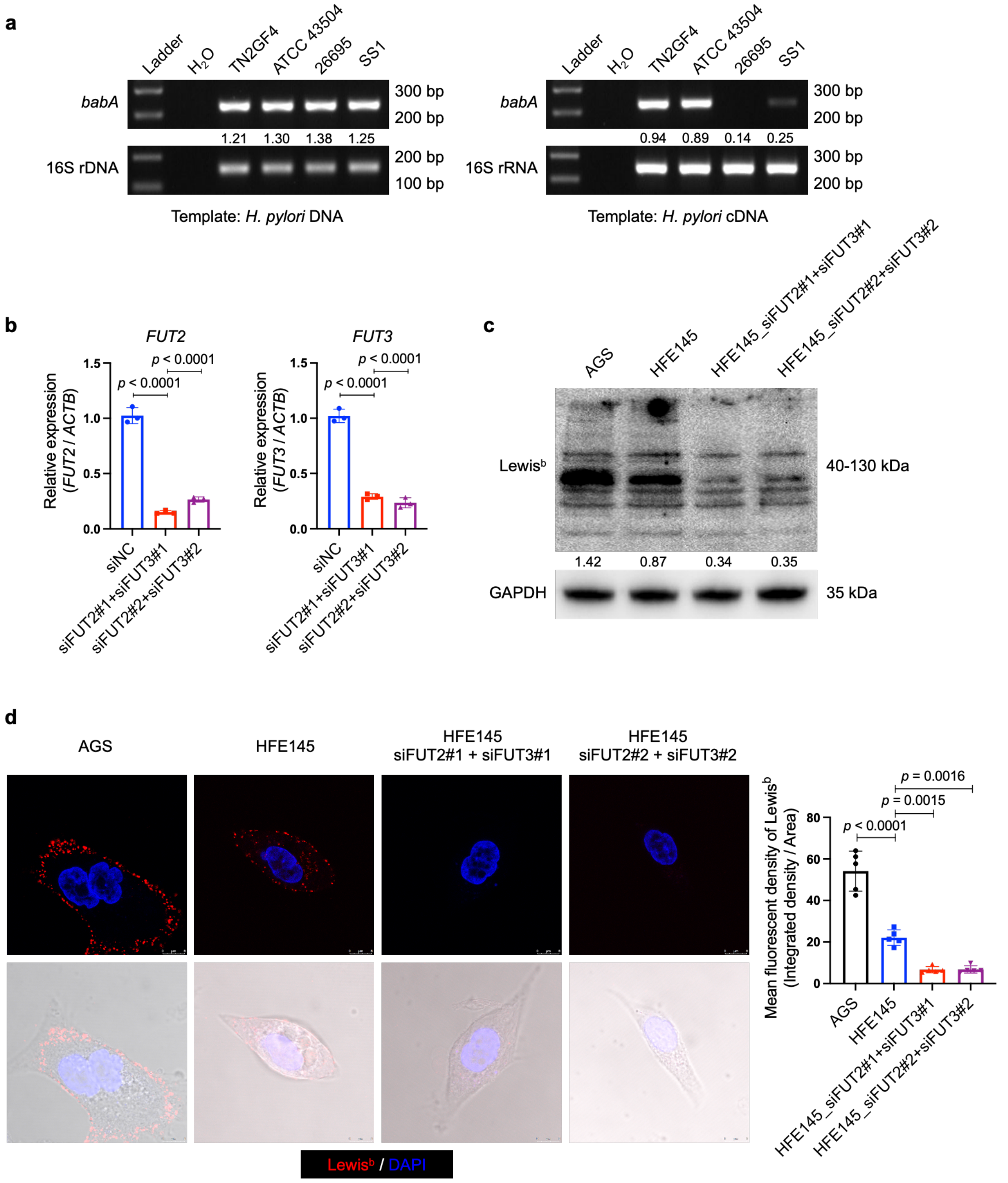

(a) Semi-quantitative PCR was performed to detect the expression of *babA* in various *H. pylori* strains. Left panel: *H. pylori* DNA was used as template to test the existence of *babA* gene in different strains. Right panel: *H. pylori* complementary DNA (cDNA) was used as template to measure the levels of *babA* transcript in different strains. **(b)** Two individual siRNAs were transfected into HFE145 cells to knock down *FUT2* and

FUT3. The knockdown efficiency was examined by quantitative real-time PCR. *ACTB* was used as an internal control (n = 3 cells for each group). **(c)** Lewis^b expression levels of each group were examined by Western blots. Human GAPDH was used as a loading control. The blots shown are representative of three independent experiments with similar results. **(d)** AGS and HFE145 cells were stained to visualize Lewis^b (red) and the nuclei (Blue). Five visual fields of each group were randomly selected to measure mean fluorescent density of Lewis^b. Scale bar, 8 μm. Quantitative data are shown as means ± SD. Statistical analysis of the data was performed using one-way ANOVA followed by Tukey's multiple comparison tests with adjustments and the corresponding *p*-values are included in the figure panels. The statistical significance of the data was calculated from one of three independent experiments with similar results. Source data are provided as a Source Data file.
