# Supplementary material for: LOX-1 acts as an N6-methyladenosine-regulated receptor for Helicobacter pylori by binding to the bacterial catalase
Source: Nat Commun. 2024 Jan 22;15:669. doi: 10.1038/s41467-024-44860-9 (PMC10803311; doi:10.1038/s41467-024-44860-9)
Supplement: Supplementary file 3 — Description of Additional Supplementary Files [file 41467_2024_44860_MOESM3_ESM.pdf]

Description of Additional Supplementary Files:

Supplementary Data 1: Mutated sites and methods for luciferase reporter assay.
