# Supplementary material for: LOX-1 acts as an N6-methyladenosine-regulated receptor for Helicobacter pylori by binding to the bacterial catalase
Source: Nat Commun. 2024 Jan 22;15:669. doi: 10.1038/s41467-024-44860-9 (PMC10803311; doi:10.1038/s41467-024-44860-9)
Supplement: Supplementary file 4 — Supplementary Data 1 [file 41467_2024_44860_MOESM4_ESM.zip › Supplementary Data 1.docx]

**Supplementary Data 1.**

GGAGGAACCCCAGCTACCCATGGCTCTGGGAGGACGGTTCTCCTTTGATGCCCCACTTATTTAGAGTCCGAGGCGCTGTCTCCCAGACATACCCTTCAGGTACCTGTGCATATATACAACGAGGAGCTGTTTATGCGGAAAACTGCATTTTAGCTGCCTTCAGTATATGTCAGAAGAAGGCAAACCTAAGAGCACAGTGAatttgaaggctctggaagaaaagaaaaaagtctttgagttttattctggaatttaagctattctttgtcacttgggtgccaaacatgagagcccagaaaactgtcatttagctggctgcagaactcctttgcagaaactggggttccaggagcctggcacctttatgtcaacatttttgattctagctacctgtattatttcacctagcttgtcccaagcttccctgccagcctgaagtccattttcccctttttattttaaaatttgactcctcttcaagcttgaaaaccctctgaactcagtcttctttacctcattatcaccttcccctcacactcctaaaattgcatgaaagacagaacatggagaacttgctcaagtgcaggcagagagcaaaaaggggaaatatgtctgggaaaaagtgcacgtgaagaaacaaagaaggacagaggccattccgaaatcaagaaactcatgttcttaactttaaaaaaggtatcaatccttggtttttaaactgtggtccatctccagactctaccacttacggacagacagacagacagacacacacacacacacacacacacattttgggacaagtggggagcccaagaaagtaattagtaagtgagtggtcttttctgtaagctaatccacaacctgttaccacttcctgaatcagttattatttcttcatttttttttctaccagaggacagattaatagatttaacccttcacaacagttcttgttagaatcatgggatgtgtggcccagaggtaagaatagaatttctttccctaaagaacataccttttgtagatgaactcttctcaactctgttttgctatgctataattccgaaacatacaagacaaaaaaaatgaagacactcaatctagaacaaactaagccaggtatgcaaatatcgctgaatagaaacagatggaattagaaatatatcttctatttttaggcttctatttcctttccacccactcttcacaggctattctactttaaaggaagcctttttattttgctgcacacaatctagcaggaatctttttttttttttaagagctgtgtcatccttatgtaggcaagagatgtttgcttttgttaaaagctttattgagatataattaacataaaataaactgaacatatttaaagtgtactatttgataagttttcacaccttgtggagaacatgcatactacaattaagagagtgaacatatccatcatccctcaaagtgtcacaatgctcctcctgatgactcctccccagaaaaccaccaatcggctttcattttgcattttgtagttttatgtgaatggaatcatatagtatgtcttttttttttgtctggcttctttcactttgcataattattttgagattcatatgtctccatcttgatgctcgtatgaattcattcttttaaatgttgaatattcccttgtatggatataccacaattcatttacccatttacttgttgatgacatttgggttgttttagttttgggatattacaaataaagctgctgtgaacatttgtgtacaag

Full-length 3'-UTR of LOX-1 (lower case) and partial CDS near stop codon (upper case) were shown above. The bases highlighted in red were the potential m^6^A motifs located in the regions of m6A peaks. Q5® Site-Directed Mutagenesis Kit (NEW ENGLAND Biolabs, E0554S) were applied to convert the adenosines (A) in m^6^A motifs to cytosine (C) according to the manufacturer’s instruction.
