# Supplementary material for: LOX-1 acts as an N6-methyladenosine-regulated receptor for Helicobacter pylori by binding to the bacterial catalase
Source: Nat Commun. 2024 Jan 22;15:669. doi: 10.1038/s41467-024-44860-9 (PMC10803311; doi:10.1038/s41467-024-44860-9)
Supplement: Supplementary file 5 — Reporting Summary [file 41467_2024_44860_MOESM5_ESM.pdf]

nature portfolio

Corresponding author(s): William K.K. Wu, NCOMMS-23-49746-A

Last updated by author(s): Dec 13, 2023

Reporting Summary

Nature Portfolio wishes to improve the reproducibility of the work that we publish. This form provides structure for consistency and transparency in reporting. For further information on Nature Portfolio policies, see our Editorial Policies and the Editorial Policy Checklist.

Statistics

For all statistical analyses, confirm that the following items are present in the figure legend, table legend, main text, or Methods section.

n/a	Confirmed	
☐	☒	The exact sample size (*n*) for each experimental group/condition, given as a discrete number and unit of measurement
☐	☒	A statement on whether measurements were taken from distinct samples or whether the same sample was measured repeatedly
☐	☒	The statistical test(s) used AND whether they are one- or two-sided<br>*Only common tests should be described solely by name; describe more complex techniques in the Methods section.*
☒	☐	A description of all covariates tested
☐	☒	A description of any assumptions or corrections, such as tests of normality and adjustment for multiple comparisons
☐	☒	A full description of the statistical parameters including central tendency (e.g. means) or other basic estimates (e.g. regression coefficient) AND variation (e.g. standard deviation) or associated estimates of uncertainty (e.g. confidence intervals)
☐	☒	For null hypothesis testing, the test statistic (e.g. *F*, *t*, *r*) with confidence intervals, effect sizes, degrees of freedom and *P* value noted<br>*Give P values as exact values whenever suitable.*
☒	☐	For Bayesian analysis, information on the choice of priors and Markov chain Monte Carlo settings
☒	☐	For hierarchical and complex designs, identification of the appropriate level for tests and full reporting of outcomes
☒	☐	Estimates of effect sizes (e.g. Cohen's *d*, Pearson's *r*), indicating how they were calculated

Our web collection on statistics for biologists contains articles on many of the points above.

Software and code

Policy information about availability of computer code

Data collection	For m6A-seq assay, both input and m6A IP samples were deeply sequenced on the Illumina HiSeq 2500 instrument. For RNA-seq assay, samples were sequenced on Illumina Novaseq 6000. RT-qPCR data was collected using Quantstudio TM 12K Flex Real-time PCR system. Western blotting, m6A dot-blotting data and agarose gel images were acquired using Biorad Chemidoc MP. Immunofluorescence images were collected with Leica confocal microscopy platform (SP8). Luciferase reporter signals were detected with Perkin Elmer Victor X4 2030 Multilabel Reader. Immunohistochemistry staining, hematoxylin & eosin staining data was collected using Olympus BX43 microscope.
Data analysis	For m6A-seq data analysis, the reads from input and m6A-IP sequencing libraries were aligned to hg19 reference genome using Tophat (version 2.0.14). Both MACS/MACS2 (version 2.2.9.1) and exomePeak (version 2.16.0) were used to call m6A peaks based on the m6A-seq bam files. The differentially methylated transcripts between groups were identified with MeTDiff R-package (version 1.1.0). For RNA-seq data analysis, the raw RNA-Seq sequence reads were trimmed using Trimmomatic (version 0.39) to remove low-quality reads and adaptors. Trimmed data were first evaluated by the software "FastQC" (https://www.bioinformatics.babraham.ac.uk/projects/fastqc/) with the default parameter and then aligned with Hisat2 (version 2.1.0) against the human (hg38) genome guided by GENCODE gene annotation (version 34) with the default parameter. The abundance of genes in each sample was calculated by StringTie packages (version 2.1.2) with the "-e" parameter. Differentially expressed genes were identified using the R package DESeq2 (version 1.34.0). Both read alignments of m6A-seq and RNA-seq on genome were visualized using IGV (version 2.8.4). Western blotting, m6A dot-blotting and immunofluorescence images were analyzed with Image J software (version 1.52a). The statistical analysis were performed using GraphPad Prism software (version 9).

For manuscripts utilizing custom algorithms or software that are central to the research but not yet described in published literature, software must be made available to editors and reviewers. We strongly encourage code deposition in a community repository (e.g. GitHub). See the Nature Portfolio guidelines for submitting code & software for further information.

Data

Policy information about availability of data

All manuscripts must include a data availability statement. This statement should provide the following information, where applicable:

- Accession codes, unique identifiers, or web links for publicly available datasets
- A description of any restrictions on data availability
- For clinical datasets or third party data, please ensure that the statement adheres to our policy

Unprocessed western blots, processed m6A-seq and RNA-seq data, mass spectrum analysis results, quantitative data from both in vivo and in vitro studies and associated statistical analysis results have been provided in Source data file. The raw data of m6A-seq and RNA-seq generated in this study have been deposited in the Gene Expression Omnibus (GEO) database under accession code GSE220810 (https://www.ncbi.nlm.nih.gov/geo/query/acc.cgi?acc=GSE220810). Human (hg19, hg38) genome data was downloaded from UCSC Genome Browser (https://hgdownload.soe.ucsc.edu/downloads.html). Source data are provided with this paper.

Research involving human participants, their data, or biological material

Policy information about studies with human participants or human data. See also policy information about sex, gender (identity/presentation), and sexual orientation and race, ethnicity and racism.

Reporting on sex and gender	The sex/ gender of human participants was determined based on self-reporting. The human participants providing samples for our study were broadly age- and sex-matched and are detailed in Supplementary Table 1 and Supplementary Table 2. Consent has been obtained for sharing of individual-level data. A nationwide survey of H. pylori investigation has showed that male sex were independent infection risk factors for H. pylori positive. However, the sex difference did not find in children and adolescent group (PMID: 36690433). Due to the small sample size, our study was not sufficiently powered to detect differences according to sex/gender.
Reporting on race, ethnicity, or other socially relevant groupings	Only Chinese patients (Nanchang and Hong Kong) are included.
Population characteristics	See Supplementary Table 1 and Supplementary Table 2.
Recruitment	See Supplementary Table 3.
Ethics oversight	The study was approved by the Research Ethics Board of the First Affiliated Hospital of Nanchang University (CDYFYYLK (01-009)) and the Joint CUHK-NTEC Clinical Research Ethics Committee (CRE-2014.133-T)

Note that full information on the approval of the study protocol must also be provided in the manuscript.

Field-specific reporting

Please select the one below that is the best fit for your research. If you are not sure, read the appropriate sections before making your selection.

[x] Life sciences [ ] Behavioural & social sciences [ ] Ecological, evolutionary & environmental sciences

For a reference copy of the document with all sections, see nature.com/documents/nr-reporting-summary-flat.pdf

Life sciences study design

All studies must disclose on these points even when the disclosure is negative.

Sample size	No statistical method was used to predetermine sample size. Sample size was determined based on common standard in research field and our previous publications (PMID: 29327342, 30612517), ensuring a minimum of n=3 biological replicates for in vitro experiments and a minimum of n=6 animals for in vivo studies with sufficient reproducibility.
Data exclusions	No data was excluded from the analysis.
Replication	For in vivo study, at least two batch animals were used and the results were successfully replicated. For in vitro experiments, the 16S rDNA PCR, immunofluorescence and colony-forming assay were performed at lease three times and the results were successfully replicated. Western-blot experiments were repeated at least twice and the results could be replicated. Other qPCR experiments and luciferase reporter assay were repeated at least twice and the results were successfully replicated.
Randomization	For BI-0115 in vivo study, 5 males and 5 females mice were randomly allocated into each group to exclude the gender-specific effects. For other animal studies, mice with same gender and genotype were randomly allocated into each group. For all in vitro experiments, samples were randomly allocated into different groups.
Blinding	Animal studies were not blinded during samples collection as the researcher need to make sure the grouping was correct. For assessment of H. pylori-induced gastric inflammation, sections were stained with hematoxylin and eosin (H&E) and scored by two experienced pathologists

who were blinded to the experiments. For in vitro assays, experiments were not conducted in blind as we performed the experiments using the same protocol.

Reporting for specific materials, systems and methods

We require information from authors about some types of materials, experimental systems and methods used in many studies. Here, indicate whether each material, system or method listed is relevant to your study. If you are not sure if a list item applies to your research, read the appropriate section before selecting a response.

Materials & experimental systems

n/a	Involved in the study
☐	☒ Antibodies
☐	☒ Eukaryotic cell lines
☒	☐ Palaeontology and archaeology
☐	☒ Animals and other organisms
☒	☐ Clinical data
☒	☐ Dual use research of concern
☒	☐ Plants

Methods

n/a	Involved in the study
☒	☐ ChIP-seq
☒	☐ Flow cytometry
☒	☐ MRI-based neuroimaging

Antibodies

Antibodies used

Antibodies used in western blotting:
METTL3 (D2I6O) Rabbit mAb #96391, Cell Signaling Technology, 1:1000
METTL14 (D8K8W) Rabbit mAb #51104, Cell Signaling Technology, 1:1000
WTAP Antibody #56501, Cell Signaling Technology, 1:1000
Recombinant Anti-FTO antibody [EPR6895] (ab124892), Abcam, 1:1000
Anti-ALKBH5 antibody ab69325, Abcam, 1:1000
Ox-LDL R-1 Antibody (LOX19-22): sc-66155, Santa Cruz Biotechnology, 1:200
CagA Antibody (A-10): sc-28368, Santa Cruz Biotechnology, 1:200
His-Tag Antibody (H-3): sc-8036, Santa Cruz Biotechnology, 1:200
Anti-catalase Rabbit pAb, constructed by Abclonal company, 1:1000
Blood Group Lewis b Antibody (2-25LE): sc-51513, Santa Cruz Biotechnology, 1:200
β-Actin Antibody #4967, Cell Signaling Technology, 1:2000
GAPDH Antibody (G-9): sc-365062, Santa Cruz Biotechnology1:200
Anti-rabbit IgG, HRP-linked Antibody #7074, 4Cell Signaling Technology, 1:5000
Anti-mouse IgG, HRP-linked Antibody #7076, Cell Signaling Technology, 1:5000

Antibodies used in m6A dot-blotting:
m6A antibody - 202 003, Synaptic Systems, 1:2000
Anti-rabbit IgG, HRP-linked Antibody #7074, 4Cell Signaling Technology, 1:5000

Antibodies used in Immunofluorescent staining:
Anti-Helicobacter pylori antibody (ab7788), Abcam, 1:200, for in vitro assay
Anti-Helicobacter pylori antibody [SPM526] (ab231433), Abcam, ab231433, 1:200, for in vivo assay
Anti-LOX 1 antibody (ab60178), Abcam, 1:100
His-Tag Antibody (H-3): sc-8036, Santa Cruz Biotechnology, 1:50
Blood Group Lewis b Antibody (2-25LE): sc-51513, Santa Cruz Biotechnology, 1:50
Goat anti-Rabbit IgG (H+L) Cross-Adsorbed Secondary Antibody, Alexa Fluor™ 488, Invitrogen 1:500
Goat anti-Rabbit IgG (H+L) Cross-Adsorbed Secondary Antibody, Alexa Fluor™ 568, Invitrogen, 1:500
Goat anti-Mouse IgG (H+L) Highly Cross-Adsorbed Secondary Antibody, Alexa Fluor™ 568, Invitrogen, 1:500

Antibodies used in immunohistochemistry staining:
METTL3 (D2I6O) Rabbit mAb #96391, Cell Signaling Technology, 1:50
METTL14 (D8K8W) Rabbit mAb #51104, Cell Signaling Technology, 1:50
WTAP Antibody #56501, Cell Signaling Technology, 1:50

Antibodies used in m6A-seq and MeRIP-qPCR:
m6A antibody - 202 003, Synaptic Systems, 1:50
Rabbit (DA1E) mAb IgG XP® Isotype Control #3900, Cell Signaling Technology, 1:50

Antibodies used in co-immunoprecipitation:
Ox-LDL R-1 Antibody (LOX19-22): sc-66155, Santa Cruz Biotechnology, 1:50
Anti-catalase Rabbit pAb, constructed by Abclonal company, 1:50

Validation	Anti H. pylori-catalase Rabbit pAb was constructed by Abclonal company and was validated using the catalase knockout strains by western blotting, as shown in Supplementary Figure 7d. All other antibodies used in this study were commercially available and were validated by the manufactures, validation statements are available on the manufacturer's website.

Eukaryotic cell lines

Policy information about cell lines and Sex and Gender in Research

Cell line source(s)	Human gastric epithelial cell line HFE145 was a kind gift from Prof. Hassan Ashktorab (Howard University, Washington, D.C., USA) and Dr. Duane T. Smoot (MeharryMedical College, TN, USA). Human gastric adenocarcinoma cell line AGS was from Prof. Jun Yu (The Chinese University of Hong Kong, HKSAR, China).
Authentication	It was not tested for cell line authentication
Mycoplasma contamination	The cells lines used in this study are tested periodically for Mycoplasma infection and were negative.
Commonly misidentified lines (See ICLAC register)	No commonly misidentified cell lines were used in this study.

Animals and other research organisms

Policy information about studies involving animals; ARRIVE guidelines recommended for reporting animal research, and Sex and Gender in Research

Laboratory animals	C57BL/6 mice, Mettl3+/- mice or Lox-1-/- mice, 6-8 weeks old, were used in this study. All mice were on the C57BL/6 genetic background. Mice were housed under 12-hour light/dark cycles in a pathogen-free room with clean bedding and free access to food and water, and temperature and humidity were kept at 22-26°C, 55%±5%.. Cage and bedding changes were performed each week.
Wild animals	No wild animals were used in this study.
Reporting on sex	For BI-0115 in vivo study, 5 males and 5 females mice were randomly allocated into each group to exclude the gender-specific effects.
Field-collected samples	No field-collected samples were used in this study.
Ethics oversight	All animal studies were performed in accordance with the guidelines approved by the Animal Experimentation Ethics Committee of The Chinese University of Hong Kong and Nanchang University.

Note that full information on the approval of the study protocol must also be provided in the manuscript.

Plants

Seed stocks	*Report on the source of all seed stocks or other plant material used. If applicable, state the seed stock centre and catalogue number. If plant specimens were collected from the field, describe the collection location, date and sampling procedures.*
Novel plant genotypes	*Describe the methods by which all novel plant genotypes were produced. This includes those generated by transgenic approaches, gene editing, chemical/radiation-based mutagenesis and hybridization. For transgenic lines, describe the transformation method, the number of independent lines analyzed and the generation upon which experiments were performed. For gene-edited lines, describe the editor used, the endogenous sequence targeted for editing, the targeting guide RNA sequence (if applicable) and how the editor was applied.*
Authentication	*Describe any authentication procedures for each seed stock used or novel genotype generated. Describe any experiments used to assess the effect of a mutation and, where applicable, how potential secondary effects (e.g. second site T-DNA insertions, mosiacism, off-target gene editing) were examined.*
